# Homelessness in pregnancy: life course factors and mental health in the context of COVID-19

**DOI:** 10.3389/fpsyt.2025.1509350

**Published:** 2025-12-26

**Authors:** Noelene K. Jeffers, C. Anneta Arno, Kelly Sweeney McShane, Makeda Vanderpuije, Andrew Lozano, Heather M. Bradford, Rebecca Shasanmi-Ellis, Karen Trister Grace, Kelley N. Robinson, Christina X. Marea

**Affiliations:** 1 Johns Hopkins School of Nursing, Baltimore, MD, United States; 2 DC Health, Office of Health Equity, Washington, DC, United States; 3 Community of Hope, Washington, DC, United States; 4 Georgetown University Berkley School of Nursing, Washington, DC, United States; 5 George Mason University School of Nursing, Fairfax, VA, United States; 6 University of Maryland School of Nursing, Baltimore, MD, United States

**Keywords:** pregnancy, homelessness, mental health, Washington DC, COVID-19, qualitative

## Abstract

**Introduction:**

Homelessness during pregnancy is a significant public health issue in the US that increases the risk of adverse maternal and infant mental and physical health outcomes. The COVID-19 pandemic exacerbated these risks through disruptions in health and social services, employment, and housing stability. Our study aimed to explore how early and cumulative adverse life experiences, mental health challenges, and the pandemic shaped the experience of homelessness during pregnancy.

**Methods:**

We used ​an action-oriented approach for this qualitative exploratory study. We conducted 20 in-depth semi-structured interviews in 2022 among a sample of pregnant, postpartum, and parenting people in Washington DC who experienced homelessness during the COVID-19 pandemic. We conducted a directed content analysis and utilized a life course perspective as the guiding analytic framework.

**Results:**

We identified six themes: early family instability - childhood through adolescence, vulnerability and conflict as an emerging adult, economic precarity during adulthood, desire for intergenerational family stability and wellbeing, impacts of COVID on homelessness and housing instability for pregnant people, and mental health and housing instability during pregnancy.

**Discussion:**

Findings highlight that homelessness during pregnancy reflects cumulative adversity which compound across the life course, with the potential to cause intergenerational consequences for maternal and infant health. Policies that ensure stable, safe housing during the perinatal period, integrated mental health care, and economic supports are urgently needed. We identify critical opportunities for policy and practice reforms, emphasizing the need for trauma-informed solutions using a life-course approach.

## 1 Introduction

Homelessness during pregnancy is a significant public health issue in the United States (U.S.) where rates are rising, especially among those aged 25-29 (1). The prevalence of U.S. pregnant people of all ages experiencing homelessness at the time of giving birth increased by 72.1% over a 5-year period, from 1 in 1314 deliveries in 2016 to 1 in 764 deliveries in 2020 (1). Homelessness describes the experience of living without a fixed, regular, and adequate nighttime residence. Homelessness is typically measured via point-in-time estimates where in public health workers canvas areas based on a geographical sampling frame and count people who are unhoused that day (2). These counts are combined with facility measures of people living in temporary or transitional shelters, and unsheltered people staying outdoors or other places not meant for human habitation (3, 4). Homelessness falls within the broader category of housing instability, a term which encompasses a range of transient housing situations such as temporary or emergency shelters, on the street, “doubled-up” (staying with family and friends), or in transitional housing (5). While this study centers on the experience of homelessness during pregnancy, we draw from the broader literature on housing instability, as many of the pathways leading to poor perinatal outcomes and adverse mental health have been studied across the full spectrum of housing instability.

Housing instability is associated with decreased access to and utilization of health care, and increased exposure to chronic and toxic stress and adverse environmental conditions, all of which contribute to worsened physical and mental outcomes for pregnant and postpartum people and infants (5–9). Adverse physical outcomes in the perinatal period (defined as the pregnancy, birth and the postpartum period up to 12 months) include preterm birth, low birth weight, neonatal intensive care unit admission, infectious diseases, excessive weight gain in pregnancy, pre-eclampsia, cardiac conditions, and extended hospitalization (1, 6, 10). Mental health outcomes in the perinatal period include substance use disorders (tobacco, illicit drugs, and alcohol) and mental health conditions, such as schizophrenia, bipolar, depressive and anxiety disorders, suicidal ideation, and suicide attempt (1, 11).Infants exposed to housing instability are less likely to receive preventative care and are at increased risk of acute and chronic health issues for at least 6 years beyond the period of homelessness (10, 12).

The COVID-19 pandemic intensified these physical and mental health risks. Between 2020-2023, the pandemic contributed to five main public health concerns in Washington D.C.: 1) delayed preventative and chronic disease care; 2) long-term effects of COVID-19 infection; 3) negative economic impact and job loss; 4) mental health stress, social isolation, trauma, and grief; and 5) loss of academic, social, and emotional growth in children (13). There was a loss of in-person community-based health care services, including perinatal home visiting (13). Concurrently, those at lower income levels experienced a disproportionately higher incidence of job loss and childcare closures, which also threatened the financial stability of many individuals and families. This is mostly attributed to the loss of many retail, service, hospitality and related sector jobs during the pandemic (13). The pandemic brought into stark relief the economic precarity of many D.C. residents.

### 1.1 Trends in homelessness

The number of people experiencing homelessness dropped between 2020 and 2021 as a direct result of pandemic-related funding (i.e., cash assistance programs) and a burst of rapid policy changes (i.e., eviction moratoriums, extended terms of subsidized housing/shelter) (14, 15). These interventions reduced the risk of financial insecurity and protected people from losing their homes. However, the number of people experiencing homelessness in 2022 climbed again as pandemic-era restrictions were lifted and eviction moratoria ended. In the national January 2023 point in time count, 653,104 reported experiencing homelessness, which was the highest since count reporting began in 2007 (4). There continue to be indirect adverse impacts of the pandemic, including increased housing costs and lack of affordable housing (16).

Similar to national trends and implementation of federal policies in housing retention and eviction moratorium, rates of homelessness in D.C. between 2020 and 2022 decreased by 30.9% with a historic 2022 low of only 7,605 people experiencing homelessness (17–19). The increased housing stability and cash support to economically marginalized families in D.C. improved key outcomes including housing stability, adult mental health, food security and child well-being (20). However, homelessness in D.C. subsequently increased 11.6% between 2022 and 2023 as pandemic-era programs began to expire, which is the data collection period for this study (16). In 2023, 34.5% of the almost 5,000 people who were homeless identified as female, highlighting the substantial proportion of people at risk for pregnancy in this subpopulation.

Homelessness in D.C. is a particularly racialized experience, with Black residents facing disproportionate disadvantage. Approximately 46% of the city’s population is Black, yet in May 2022, Black residents comprised 86% of people experiencing homelessness (21). Disparities in housing instability in D.C., particularly for Black residents, reflect complex historical racial inequities in the city (22). Structural racism, defined as macro-level infrastructure and policies that reduce access to opportunities, resources, and power, contributes to racial inequities in housing stability and homelessness (23–25). Over the past 20 years, D.C. has weathered immense economic change, and pursued an intense urban renewal plan and thus attracted a market driven investment. However, this rapid and uneven investment resulted in gentrification, displacement of long-time Black residents, community disinvestment, and limited access to affordable housing (26–28).

### 1.2 Mental health and pregnancy

Pregnancy can be a stressful time for the birthing person in the general population, including concerns of low material resources, poor social support, work/family responsibilities, and pregnancy complications (29). Stress and anxiety are quite common in pregnancy. Depression and anxiety were worse among all U.S. persons during the pandemic. Multiple U.S. studies found higher levels of depression and anxiety compared to pre-pandemic rates, increasing from 13-16% to 36% for depression and 0.3-5.3% to 22% for anxiety (30). Stress and mental health conditions in pregnancy can negatively impact pregnancy experiences and perinatal outcomes (31).

### 1.3 Adverse factors associated with homelessness and pregnancy

Homelessness during pregnancy poses several risks to perinatal health, with research pointing to a complex web of contributing social, economic, and environmental factors. The D.C. Maternal Mortality Review Committee has identified challenges in accessing safe and stable housing throughout the lifespan as a contributing factor to some maternal deaths (32). Qualitative research on the lived experience of homelessness during pregnancy is limited; however, existing literature describes the persistent relationship between several factors or conditions that occur when a pregnant person lacks housing stability: experiences of violence and abuse, economic vulnerability, mental health challenges, and substance use (5, 33). Most significantly and consistently reflected in the literature is the prominent role of violence and abuse among pregnant homeless women, either leading up to the period of homelessness (33–35), or as an extreme vulnerability during homelessness, or both (34–36). Mental health disorders and substance use are closely associated with and contribute to homelessness and pregnancy complications (33, 35–37). Separate from the above challenges, those experiencing homelessness during pregnancy also experience significant stigma from healthcare providers, family members, and others, limited access to health care services, including prenatal and mental health care (33–37), as well as an increased exposure to environmental contaminants (38, 39).

### 1.4 Perinatal mental health and homelessness

Mental health conditions are the foremost underlying cause of maternal mortality in the U.S., responsible for 23% of all pregnancy-related deaths, including deaths from suicide, overdose, and substance use disorder (40). Analysis of mental health conditions in the perinatal period must consider social determinants of health (SDOH) factors as risk factors for adverse health outcomes and maternal mortality (29). Despite a historical focus on individual health behaviors as critical determinants of increased maternal morbidity and mortality, research in recent years has examined a broader context of SDOH, defined as “nonmedical factors that influence health outcomes” (41, 42). These SDOH factors include living and working conditions, food and housing security, and economic status in pregnancy, which has received growing attention in recent years (6).

### 1.5 The lifecourse perspective

A life course perspective (43) is increasingly used to understand how early-life exposures and cumulative disadvantages contribute to housing instability and homelessness (44, 45). This approach emphasizes that a person’s health status is the result of the complex interplay between the context of their lives including SDOH such as environmental exposures, social connectedness, family influences, structural marginalization, intersectional identities, and socioeconomic conditions that impact health behaviors, and genetic and epigenetic factors across the life course (46–48). This interplay begins with early-life (including fetal and childhood) exposures, and when subject to cumulative adversities, one’s health can be negatively influenced over the lifespan due to repeated stressors on the allostatic systems (49). Among these exposures, adverse childhood experiences (ACEs), including childhood homelessness, abuse, parental incarceration, witnessing violence, and/or community traumatic exposure, are particularly strong predictors of adverse mental and physical health outcomes as well as later homelessness (30). In the context of homelessness during pregnancy, a life course perspective highlights a logical pathway from cumulative adversity, housing insecurity to psychological stress, which in turn contributes to adverse perinatal outcomes (50). However, to our knowledge, homelessness in pregnancy has not been examined comprehensively using a life course perspective.

### 1.6 The current study

This article emerged from a larger qualitative study conducted as part of the D.C. Calling All Sectors Initiative (CASI), a philanthropically funded initiative led by the D.C. Health Office of Health Equity (OHE) in collaboration with Community of Hope (a federally qualified health center and homelessness service provider in D.C.) that sought to generate solutions to address structural inequities in housing (51). The broader study aimed to understand the lived experience of navigating homelessness assistance while pregnant to rapidly inform local policy and practice (9). While that study explored a range of structural and service-related challenges, this paper focuses more narrowly on the life course exposures that contributed to the concurrent experience of pregnancy and homelessness. Guided by a life course perspective, it explores how early and cumulative experiences, mental health challenges, and the COVID-19 pandemic contributed to and shaped the experience of homelessness during pregnancy.

## 2 Methods

### 2.1 Study design

This was an action-oriented qualitative exploratory study guided by a constructivist epistemology and a life course perspective (52). We used an action-oriented approach in collaboration with two community partners to generate findings aimed at driving change, with the goal of improving housing and homelessness policies for pregnant people in D.C. The D.C. Health Office of Health Equity (OHE) and Community of Hope were actively involved throughout the research process. They co-developed the interview guide, provided critical insights on preliminary themes during data analysis, and helped shape the framing of key findings and implications.

A qualitative exploratory design was well-suited to this study since it is typically employed for investigations of topics which have not previously been studied in-depth. A life course perspective informed the interview guide design, coding and thematic analysis, and identification of themes. The study was approved by the Georgetown University Institutional Review Board (#0004530).

### 2.3 Participants and recruitment

The study population included women 18 years of age or older who experienced homelessness during or within 3 months of the end of a pregnancy. Participants could be currently pregnant, within 2 years postpartum, or parenting, defined as more than 2 years since their last birth. Eligible participants were required to have sought homelessness services in D.C., speak English, and have access to a phone or computer.

We used a purposive sampling strategy to identify eligible participants by partnering with D.C. homelessness services agencies who shared study information with them. (See **Supplementary File S1** - Agencies Contacted). Interested people were able to enroll in the study and complete the informed consent process either via the study website or phone with a member of the study team. Enrolled participants then scheduled an interview and were emailed a copy of the study information. Participants received a $75 retail gift card in appreciation of their time.

### 2.4 Data collection

Interviewers used a semi-structured interview guide (See **Supplementary File S2** – Qualitative Interview Guide). We conducted in-depth semi-structured individual interviews between June and July 2022. In-depth interviews are an ideal method for inquiry into sensitive and taboo subject matter with marginalized groups to ensure privacy and explore topics most important to the participant (53). The two interviewers (first and last authors), respectively Black and Latina, are PhD-prepared nurse-midwives who identify as women, and each gave birth within the last 5 years. Both interviewers have clinical experience caring for pregnant and postpartum people experiencing homelessness, and extensive training in the conduct of qualitative research. We conducted interviews by phone or secure video conferencing (Zoom) at a time convenient to the participant.

Interviewers used a semi-structured interview guide informed by a life course perspective (See **Supplementary File S2** – Qualitative Interview Guide). Interviews began by presenting the aim of the study, reiterated that participation was voluntary, and they could withdraw at any time from the study. Interviewers explained the nature and purpose of the study prior to consent, then affirmed participants’ understanding of the study and confirmed verbal consent to proceed and audio-record. The interviews began by asking participants to share how they came to be unhoused and what it was like to experience homelessness during pregnancy or the postpartum period. The next set of questions explored what types of support they would have liked to have received, including help securing housing. The interview guide then explored strategies that the participant used to try to meet their needs while experiencing homelessness during a pregnancy, as well as their experience seeking health care during the perinatal period. The interviewers used probes to explore areas and confirm concepts that arose, especially related to barriers and facilitators of accessing support. The interviews ended with a series of demographic questions to contextualize their lived experiences. Interviews lasted 45–90 minutes and the recordings were transcribed by a professional human transcription service, and error checked.

### 2.5 Analysis

The interviewers met weekly during data collection to review content and compare themes. The interviewers determined that saturation had been achieved at interview 16, and conducted four additional interviews to ensure no new themes arose. The interviewers uploaded the interview transcripts to Dedoose, a web-based qualitative data analysis platform. The first and last authors performed the analysis using an action-oriented directed content analysis approach and inductive coding (54, 55).

The authors used content analysis and inductive abstraction to identify overarching themes that addressed the purpose of the study. We began by reading all interviews, thus becoming immersed in the data. One author created an initial codebook by inductive coding of a single transcript. A second author then applied that initial codebook to the same transcript, adding codes as needed. The authors met to review, compare applications, and clarify the codes. The authors identified main categories and subcategories of codes, and created associated summaries and applied anchor codes. Next, both authors independently applied the codebook to two more transcripts and met to review code application until >80% consensus was achieved. The authors then applied the finalized codebook to all remaining transcripts and confirmed that no new themes emerged within a single interview to ensure data saturation.

## 3 Results

### 3.1 Participant characteristics

We conducted 20 in-depth individual interviews (see **Table 1**). All participants identified as Black or African American women, and age ranged from 19 to 35 years old. A few were currently pregnant (15%, n=3, pregnant), most had birthed within the last two years (n=13, postpartum), and some had given birth more than 2 years ago (n=4, parenting). More than half (n=12) were unemployed. Roughly a third of participants first experienced housing instability in childhood (n=6) and more than half first experienced housing instability as an emerging adult (n=11).

**Table 1:** Characteristics of participants.

Characteristic	% (N)
Race - Black/African American	100% (20)
Current Age, Mean (Range) - Emerging Adult (18-24) - Adult (25+)	25.7 (19 - 35) 55% (11) 45% (9)
Pregnancy/Postpartum/Parenting Status - Currently Pregnant - Postpartum (birthed within the last 2 years) - Parenting (more than 2 years since last birth)	15% (3) 65% (13) 20% (4)
Employment Status - Part time - Full time or student - Unemployed	25% (5) 15% (3) 60% (12)
Age at First Unstable Housing - Childhood (<18) - Emerging Adult (18 - 24) - Adult (25+)	18.4 (4 - 33) 30% (6) 55% (11) 15% (3)
Education - Some high school - High school - Some college - Vocational Training - Associates	10% (2) 40% (8) 40% (8) 5% (1) 5% (1)
Relationship Status - Single - In a relationship - Engaged - Situationship - It’s complicated	35% (7) 30% (6) 5% (1) 10% (2) 20% (4)

### 3.2 Themes

We present findings which explore life course stages during which participants experienced homelessness or housing instability (Themes #1-4) and the ways in which mental health challenges and the COVID-19 pandemic contributed to and impacted experiences of homelessness while pregnant (Themes #5-6). Participants described 4 periods of vulnerability during the life course that contributed to experiencing homelessness during a pregnancy: 1) early family instability - childhood to early adolescence, 2) vulnerability and conflict as an emerging adult, 3) economic precarity during adulthood, and 4) desire for intergenerational family stability and wellbeing. Themes related to 5) the impact of the COVID-19 pandemic and 6) mental health and housing instability during pregnancy were pervasive throughout the interviews. Participant quotes include a pseudonym (created via random name generator) and their age.

#### 3.2.1 Theme #1: early family instability - childhood through adolescence

For some participants, homelessness and housing instability were a pervasive part of their lives during childhood and adolescence. Often the housing instability occurred in a family environment where other issues such as substance use, death or incarceration of a parent, and physical and sexual abuse were also present. Some women who experienced abuse spent time in the foster care system and moved around among various family members, presenting additional forms of housing instability.

*I lived with my dad as a kid. Then, I ran away [at 13] because it was toxic and … abusive. Ended up in foster care, two foster homes and a therapeutic group home. After that, my aunt got custody of me for two years … That didn’t work out. Her kids were on drugs and they were stealing from me and I experienced lots of problems there … I did a lot of house hopping. Went with my grandma actually. That didn’t last too long … We didn’t leave on bad terms but it was overwhelming for her. I went back with my dad … His wife and I have always really bumped heads and [it was] toxic. They’re still alcoholics … Anyway, long story short, she wrote me up an eviction notice and kicked me out. Again, homeless … [I was] 19. - Imani, postpartum, 24yo, age 13 at first housing instability*

Some participants experienced parental incarceration or their own juvenile incarceration, often while alienated from their family of origin, leaving them without a safety net. Participants who experienced housing instability from a young age felt they lacked adult oversight and companionship throughout their lives.

*I’ve had a juvenile record. I stayed in a detention center group home, or something of that nature, most of the time until I turned 18. Once I turned 18, I got myself together and I started working and then that’s when I found out I was pregnant … I would just say more so just not having any type of guidance once you turned 18. That is what it was for me. I didn’t have a home prior, so once I turned 18, I was just out there. - Aissatou, postpartum, 21yo, age 18 at first housing instability*

The lack of a stable parental figure upon whom they could rely meant that as they transitioned into adulthood, some relied on romantic partners for care, emotional connection, and housing. These relationships were often precarious, and upon ending, resulted in more housing instability. Several participants reported that these relationships were also characterized by intimate partner violence (IPV) and substance use.

*I never had a … good mother parent. I always had my father and stuff. When I was 16, I met my baby father and started dating him. My mom had made up so many lies … She pretended that she wanted to come get me. When my baby father had open arms for me … but my mom, she just would shut everybody down … It’s like she was willing to do anything to try to make my life bad. - Tamia, parenting, 24yo, age 16 at first housing instability*

#### 3.2.2 Theme #2: vulnerability and conflict as an emerging adult

For most study participants, the first experience of housing instability occurred during emerging adulthood (defined as age 18-24). Relationships with parents that were strained during late adolescence became more fraught as parents saw their children as adults and no longer felt responsible for housing them (what participants referred to as being “put out”). Often there was a precipitating event, like a fight or altercation that became physical. Being put out was a painful experience, and left many homeless. Participants often found out they were pregnant shortly after this.

*I didn’t know that I was pregnant until probably two weeks after I got put out [by my mom] … It wasn’t the first time they’ve done something like that before … Sometimes I couldn’t find nowhere to go … mom had just put me out … it was a serious altercation, it turned into a legal situation … and I got arrested … It hurts. – Melanie, postpartum, 24yo, age 16 at first housing instability*

For others, the precipitating event was a split from a partner on whom they were dependent for housing. Some described moving in with partners after being put out by their family of origin. The relationships often included infidelity, abuse, or abandonment that contributed to feelings of hopelessness.

*I was in the military, and then when I got out, I met a dude from DC. I moved up here with him. I would have said six months that we’ve been up here, we broke up, and I became homeless … that’s when I found out I was pregnant with my daughter … I was 20 years old … The breakup was because he was cheating. When we broke up, that’s when I found out I was pregnant. - Ciara, postpartum, 25yo, age 20 at first housing instability*

The participants who experienced housing instability as a young adult often described “couch surfing” (temporarily staying in spare, available spaces with friends and family members) as their main strategy for shelter. However, this solution was fraught with tension about length of stay, household contributions of money or food, and discomfort with drugs, alcohol or other people in the home. Participants emphasized that having temporary shelter or staying with someone they felt was “toxic” does not equate with being housed.

*When I was 18, I would stay in my mother’s house at the time. I was also in college. I wasn’t getting no money and my mom was getting extra tired of me. So she wasn’t trying to get me no money … I had went to my cousin’s house … Then she had put me out … I moved in with my cousin’s house on my mother’s side of the family. Then, her house got crowded and I didn’t have nowhere to go. I went to my dad’s house, but I slept on the floor. Then after that, my dad moved to North Carolina. I didn’t want him to go … Then when I was pregnant, I was staying over at my sister father’s house, then … I just basically rented an apartment from my mom’s old friend … [but] she kicked me out with no 30-day notice. - Brooke, postpartum, 23yo, age 18 at first housing instability*

Participants experienced significant anxiety “couch surfing” during pregnancy because they did not feel they could bring an infant back to these environments. This resulted in deep grief that they did not have family support.

*… me and my mom, we just didn’t really get along. We couldn’t be in the same house with one another. My baby, when I found out that I was pregnant with him, he had a heart disease, so it pushed me further away from my mom, because they didn’t understand the heart disease and things that was going on with my son … I was actually put out right after I had my son … he was hospitalized for his whole life. He passed away when he was 11 months. - Kennedy, pregnant, 19yo, age 16 at first housing instability*

Participants describe not knowing when, where or for how long they will be housed as immense sources of stress. The pervasive feelings of instability made it difficult to find work, care for themselves and their pregnancies, and left them with a persistent sense of hopelessness and insecurity. These feelings were heightened by fears regarding their pregnancy and worry about how they would care for their infant.

#### 3.2.3 Theme #3: economic precarity during adulthood

Some participants experienced homelessness for the first time as adults (over age 24) due to unexpected events like job loss or financial strain, separation from a partner (sometimes related to IPV), and formal or informal evictions during the COVID-19 pandemic. Sometimes the precipitating event was pregnancy related, such as termination of employment due to lack of pregnancy protections, relationship strain due to pregnancy, or landlords not allowing an additional person in a unit.

*…[my mom and I] were in a rooming house. I had gotten pregnant, and then I had to end up telling the landlord like, “Hey. I’m pregnant. This is what’s going on. There is about to be another kid in the house.” She [said] “Okay. Well, you and your mom are going to have to find somewhere else to stay because I can’t have you bring another kid in here. - Sanaa, postpartum, 24yo, age 21 at first housing instability*

For some, homelessness was linked to the financial and caregiving strain of parenting. Many participants were unable to find jobs that fully supported their families, or offered sufficient worker protections to care for themselves during pregnancy or their children.

*I used to cut this old lady’s grass … to wash super cars, take their trash out. [When], I was pregnant, they did not want me to. But I didn’t mind lifting heavy things, honestly, because I needed the money. - Ciara, postpartum, 25yo, age 20 at first housing instability*

Economic vulnerability and lack of social safety nets, coupled with life course exposures to traumatic events, such as experiencing or witnessing violence, incarceration, can contribute to mental distress. These stressors make navigating the cognitive demands of complex social services requirements feel insurmountable, especially during transitional periods, such as moving in and out of housing.

*I had been homeless before having my third son. I was in a program for women and children in DC. Then, I got arrested. I saw someone get killed and I was afraid to tell someone so they had me go through a lot of therapy for that. I went through community connection for that therapy, and then I also did probation. When I came home from jail, I was pregnant with my son … they released me to the halfway house. I stayed there, I started looking for work, doing what they asked me to do there. - Kay, postpartum, 32yo, age 4 at first housing instability*

Many participants ascribe a lack of material resources (e.g., cash savings, property ownership, or family safety net) as reasons why a precipitating event resulted in homelessness. One participant described how they were unable to work because of responsibilities as a caregiver and the strain of waiting to see if they were eligible for public assistance.

*I wasn’t working, I couldn’t work. I didn’t want to work because I had my son, and then he was going through [medical problems] … I had to be there with him. Then I got pregnant again. I didn’t want to work because I was pregnant with twins. By that time … I wanted to do work and things like that, I had to just wait for … TANF and food stamps. - Kennedy, pregnant, 19yo, age 16 at first housing instability*

The lack of affordable childcare options further drove families into poverty and housing instability. Participants described immense strain trying to find ways to work to support their families but being unable to do so.

*Since I never had a daycare voucher, it was always hard to get somebody to watch my daughter … I will work at least five days a week, sometimes more, more in the summertime … teenagers were out of school, so I had a lot of babysitting options … [but] when everybody went back to school and back to work, I could only work two days a week … Then, I was denied my voucher because they said I had to work at least 40 hours … [but] the whole point of the voucher is to get my child in daycare so I can work! I didn’t understand that. - Mia, postpartum, 19yo, age 18 at first housing instability*

These stories highlight how narrowly many participants walk the line between stability and homelessness, and the importance of ensuring that employment opportunities provide a living wage and worker protections not only for their own stability, but to make an intergenerational shift for their children.

#### 3.2.4 Theme #4: desire for intergenerational family stability and wellbeing

Participants expressed a deep desire to break cycles of instability and create a foundation of security and wellbeing for their children. Many described pregnancy as a pivotal moment that heightened their motivation to seek stable housing, strengthen family relationships, and build a better future not only for themselves but also their newborn. This desire for intergenerational stability was often shaped by participants’ own histories of childhood trauma, housing insecurity, and fractured family connections. The prospect of parenting while homeless brought fears of repeating harmful patterns, alongside hopes for providing their children with a more stable and nurturing environment than they had experienced. This participant shared:

*When I birthed that kid, it was just amazing like, “Wow. I did this. I got a reason to live now. I can’t fail this kid the way my parents failed me.” I think that one line though, that helped me the most. I always put that into my head. Anytime I get super down I just remember like, “It’s not a choice now. I cannot fail my kid like my parents failed me. I refuse.” I think that’s what just keeps me going. - Tamia, parenting, 24yo, age 16 at first housing instability*

A few participants reported a lack of services and support for their baby’s father, and how men, particularly young Black men, often struggle with housing, family strain, instability, and lack of social support. This was described in the context of wanting the baby’s father to have his own needs met, but also often needing to maintain an independent living space in cases of strained relationships, substance use, or IPV. Participants also recognized that when fathers faced instability, it often undermined the broader stability of the family unit. Housing insecurity, unemployment, or untreated trauma experienced by fathers contributed to relational strain, limited co-parenting opportunities, and increased the emotional and logistical burdens on pregnant people. As one participant explained, the absence of meaningful supports for fathers can create a myriad of unsafe and unsustainable home environments:

*Sometimes the shelter only allows the mother and the baby to come in … that’s not right either, because fathers go through things too. There’s single fathers out here … on the streets … Everybody needs help … If a mother and father’s in the house together, and they’re not on good terms, or the mother wants the father to leave, the father ain’t leaving… ‘I ain’t got the help, so I’m going to stay here.’ Then, domestic violence, that’s where it comes … that’s just a bad, toxic situation, a bad environment for the kids to be around … The fathers don’t really get the help - Jayda, pregnant, 26yo, age 18 at first housing instability*

Many voiced frustration that the short-term, fragmented housing options typically provided through the DC housing system undermined these goals. Participants described a constant cycle of temporary placements that forced them to search for new housing shortly after moving in, disrupting their ability to establish routines and foster a stable home environment for their children. This participant noted:

*I was able to move from [the shelter] into my Rapid Re-Housing [but now] I’m at the end of my [term] for Rapid Re-Housing … I’ve already signed paperwork with my case managers to end case management services because I’ve been told I’m at the end of my extension. I’m only allowed one extension in this community program, which they extended because of COVID-19… I’ve already been accepted to this program called DC Flex. This is some sort of pilot program … I’ve been concerned about not being able to pay my rent, and they’re telling me, “You know we’ll contact someone,” and I can’t contact anyone because no one returns my phone calls … I’m kind of confused as to how things are going to go moving forward … I’ve also been offered a waitlist for Section 8 through this apartment complex … I’ve been told multiple different stories about how dangerous that neighborhood is. Kay, postpartum, 32yo, age 4 at first housing instability*

Participants consistently described a desire to establish employment with enough flexibility to meet their financial needs, care for their children, and have a safe and stable home in which to raise their family.

*Man, I’ve had so many opportunities to get work. Wait. Job interviews have been set up and stuff like that, but it hasn’t worked out … I [haven’t] made it to the interview, because “Oh, I don’t have … my clothes today, I don’t have the outfit that I wanted to wear,” or just not being mentally ready. “My hair isn’t done. I don’t feel good about myself today.” - Makayla, parenting, 34yo, age 18 at initial housing instability*

At the time of these interviews, some of the COVID-era policies extending eviction moratoriums and increased rental and cash assistance were set to expire. During the COVID-19 pandemic, it was increasingly difficult to find childcare. Without stable childcare, many found it difficult, if not impossible, to maintain work outside the home. There was pervasive anxiety expressed from the participants that they would soon be homeless again.

*With COVID going on, they can’t expect people to find jobs and have things in order. There was a whole pandemic for almost two years! There’s no way that people can just go start paying rent on their own and just living on their own with no jobs and stuff … They were extending [housing support] because of COVID, but now they just said it’s over in April. No more extension. That’s it. That’s all. - Jayda, pregnant, 26yo, age 18 at first housing instability*

Participants felt that COVID-19 extensions provided temporary stability but were not sufficient to lay a foundation for long-term stability, particularly given the economic precarity of the time.

#### 3.2.5 Theme #5: impacts of COVID on homelessness and housing instability for pregnant people

Participants were deeply appreciative of the extensions and flexibility in housing support that were provided during the COVID-19 pandemic, particularly in light of increased difficulty finding stable employment with sufficient income and flexibility to support parenting. This study occurred in June-July 2022, a time during which many COVID-19 exceptions were expiring, and many shared fear and anxiety about what would happen once the support was gone…*. [housing support] extended because of COVID-19. My six months will come to an end on April 30th of this year. I’ve already been accepted to this program called DC Flex…. It’ll [give me] about $8500 a year so up to about $700 each month towards my rent…. I’ve been concerned about not being able to pay my rent … I’m kind of confused as to how things are going to go moving forward in April. - Kay, postpartum, 32yo, age 4 at first housing instability*

During the COVID pandemic, it was increasingly difficult to find childcare. Without stable childcare, many participants found it difficult, if not impossible, to maintain work outside the home. Often childcare hours did not align with work hours, and without protections for parents and caregivers, participants reported being unable to keep their jobs.

*… with COVID going on [my daycare is] short-staffed, they used to be 6:00a to 7:00p … but now you can’t drop off earlier than [830am] and you have to pick them up by 5:00… It’s very hard to find [employers] that are even willing to work around that…. I found work, and then boom, they fire me because of this– Anyway, sleeping in the truck, it was hard when it was cold trying to cut on the heat and all and just still being scared of … violence and just scared to go and sleep. - Imani, postpartum, 24yo, age 13 at first housing instability*

Pandemic-era changes to public transportation schedules were also mentioned as challenges that adversely impacted their ability to work. For those who were pregnant and living in group shelters during pregnancy, there was the additional fear of becoming sick with COVID given the close living conditions. Other participants in shelters gave up their jobs during the pandemic so as to avoid infecting other people in the shared spaces, further exacerbating their financial precarity.

*I got my [Inclusionary Zoning] certificate … but during the [COVID-19] pandemic, I had to stop working because the public transportation closed down … You going to end up back in the streets again after the year when they are done helping you … I worked November 2019 to like June 2020. I was working till my ninth month in the pregnancy and the whole COVID thing came and they shut down. Since I was living in a maternity home, I couldn’t also work in case of catching a disease and losing the home. - Asia, postpartum, 23yo, age 21 at first housing instability*

Participants who were pursuing housing support during the COVID-19 era reported increasing delays and lack of communication from housing services. The intersecting challenges of housing instability, pregnancy, economic precarity, and fear of COVID created immense anxiety and insecurity.

*Well, I would say the only barrier is really COVID, this whole pandemic stuff, because it slows the process and then everything was shut down or people weren’t accepting things right now. I tried to apply for Section 8 again recently, and their list is closed [due to COVID] so I can’t do it. - Mia, postpartum, 19yo, age 18 at first housing instability*

#### 3.2.6 Theme #6: mental health and housing instability during pregnancy

Participants described homelessness during pregnancy as stressful and scary, and many felt that being homeless undermined their ability to feel like a good parent. Some had lifelong struggles with depression and anxiety that contributed to homelessness. For others, it was the homelessness that spurred the development of mental health challenges, and their basic needs for safety and survival were not being met.

Participants emphasized the immense anxiety they experienced as they sought safe and stable shelter for themselves and their newborn. They described near-constant fear when they did not know if they would have a place to stay, or if they were unsure how long they would be able to stay in a current shelter or housing environment.

*… [It was] stressful because I was about to have a baby and I don’t want to be all over the place with a baby … I just had to make sure I had to find some [place to live] so when my mom’s friend let me rent the apartment from her. I thought everything was fine. I felt great but when she kicked us out, my depression was so horrible. - Brooke, postpartum, 23yo, age 18 at first housing instability*

For a few participants, hopelessness led to struggles with suicidality.

*The only reason why I was feeling suicidal was, honestly, because I didn’t have any support, any family, or the child’s father wasn’t supportive at all … I needed help … it would get very, very overwhelming to where I’m just like, “I’m ready to end it.”… Taking care of a child. We’re barely making it … like, damn, I don’t get no break … it was just taking me into a depression … a lot of times I be like, “If I just end it, everything will be okay,” but in reality, it won’t be okay. - Ciara, postpartum, 25yo, age 20 at first housing instability*

Participants described fear related to their safety when sleeping on the street where they feared violence, theft, and injury to themselves or the fetus. They also described concerns regarding exposure to severe weather conditions, including cold weather in winter and heat in summer. Participants reported fear in many of the shelters they utilized, including public shelters and homes of friends and family, such as concerns of theft or attack from other residents, particularly when they were required to stay in shared spaces. This participant describes the fear she felt sleeping in her car, which was broken into one cold winter night:

*Sleeping in the truck, it was hard when it was cold trying to cut on the heat and all. Someone broke in my truck when I was pregnant with just my first son once. I was scared of that violence and just scared to go and sleep because I don’t know what’s going to happen or anything. - Imani, postpartum, 24yo, age 13 at first housing instability*

For many participants, mental health challenges were debilitating, making it difficult to conduct activities of daily living, manage the pregnancy, and take the necessary steps to secure long-term housing. While some participants were connected to a mental health therapist or support program, others reported no mental health support and relied on constructive and destructive (e.g., substance use) coping strategies.

*I did stress a lot. I was in the hospital because her heart rate was always going up for no reason because they was like I was stressing too much … It’s just that I just had to stop stressing because they said it’s not good for the baby and I was just like it’s so hard to do that when life is not great at all. – Brooke, postpartum, 23yo, age 18 at first housing instability*

The experience of homelessness during pregnancy often felt like part of an unrelenting series of challenges, losses, and feeling let down.

*My baby daddy, he left me when I was four months pregnant. It was just challenging all the way around from day one to losing my job, my place, my sense of security, my mental was terrible. I was just depressed. I wasn’t happy. - Samara, postpartum, 32yo, age 30 at first housing instability*

Despite the challenges that the participants faced, many shared how central their children and role as parents were to their self-worth and well-being. They shared strong desires to improve their mental health, and circumstances to create a stable environment for their child.

*It’s way easier [since COVID] with everything being virtual … [but] it’s even way harder with three kids because my mental is not there yet. It’s just overwhelming … I’ve experienced a lot of trauma and … I literally didn’t expect to live this long. I hated my life. Here I am, three kids later trying to still find my way … I don’t know how to find the time to build myself and learn about myself … I’m a great mother, but other than that, I don’t know what I’m here for … I’m struggling to try to figure it out. - Imani, postpartum, 24yo, age 13 at first housing instability*

Participants described a constant state of being overwhelmed while trying to manage the intense demands of meeting basic needs for housing and food, trying to be a good parent, find work, and maintain a sense of themselves.

## 4 Discussion

Findings from this study offer new insights into the experiences of pregnant people experiencing homelessness during the COVID-19 pandemic and frames these experiences through a life course perspective. Our findings suggest that homelessness during pregnancy may be shaped by adverse events that occur across the life course, and can contribute to profound mental health challenges. Rather than a sudden or isolated event, for participants in this study, homelessness during pregnancy emerged as a result of intersecting adversities that had compounded over time, often beginning in childhood and persisting through adolescence and adulthood. The COVID-19 pandemic created additional opportunities and stressors - extending some supportive housing services that provided short-term relief while also intensifying housing instability, particularly as early adaptive policies were discontinued. Participants experienced significant mental health challenges as they struggled to maintain a health pregnancy while lacking safe and consistent shelter. We discuss these findings and present key practice and policy implications that emerged from the data analysis.

### 4.1 Origins and sustaining factors of homelessness and lifecourse implications

Our findings are consistent with the broader literature on homelessness which describes how early life adversity and structural factors contribute to and sustain homelessness over time (44, 56). Our findings extend the existing literature, confirming that these pathways also affect pregnant people experiencing homelessness, and highlight the value of applying a life course perspective to this vulnerable population. Participants in this study report profound stressors throughout their lives, including foster system involvement and lack of safety and stability in childhood, IPV and housing instability during emerging adulthood, job insecurity, and lack of childcare throughout adulthood. Participants’ narratives revealed how cyclical patterns of instability often persist across developmental stages, with few opportunities for recovery or stability. This study reinforces prior findings that early exposure to homelessness and other ACEs contribute to ongoing housing instability across the life course.

### 4.2 COVID-19 pandemic

Our findings illuminate how the COVID-19 pandemic represented a “stress test” for marginalized people and the local and national social services infrastructure. The pandemic created additional stressors for those experiencing homelessness including fear of infection in shelters and crowded living spaces, slowed social support services, and loss of work, childcare and transportation. However, the pandemic era was also a time of increased policy experimentation that aimed to mitigate the negative economic impacts of the pandemic with eviction moratoriums, cash assistance programs, housing and social program extensions, and eased administrative burdens to maintain insurance (14, 15). These policy responses demonstrated that local and national governments can effectively address homelessness and housing insecurity with innovative programming. Structural and policy changes during the COVID-19 pandemic contributed to housing instability for many, but also led to policy experiments that resulted in increased stability for pregnant people in places that provided robust support.

### 4.3 Mental health challenges

Building on existing literature documenting the mental distress associated with homelessness (24), our participants similarly described significant grief, anxiety, and stress during the perinatal period. However, participants in this study experienced distress because the demands of maintaining a safe pregnancy were fundamentally incompatible with the realities of homelessness. Pregnant people experiencing homelessness have a brief period of time to establish a safe and stable environment for an infant, while simultaneously striving to achieve a healthy pregnancy. Managing these transitions is appreciably more difficult in the absence of stable housing and safe living conditions, thus amplifying stress and undermining opportunities for positive physical and mental health outcomes for the pregnant person and their infant. The fear and insecurity of not knowing what comes next, or when they will have housing, adversely affects participants’ mental status and ability to manage their health and pregnancy. Similar experiences are reported among people experiencing homelessness while managing chronic health conditions, undermining their ability to care for themselves, access and attend health care visits, thus threatening their overall health and wellbeing (57).

### 4.4 Structural barriers

D.C. reflects broader national trends, illustrating how structural replication of intergenerational poverty and housing instability intersect to further marginalize vulnerable parents. Historical policies and intersecting structural burdens in D.C., such as redlining, predatory lending, lack of affordable housing and under- and unemployment, have perpetuated racial inequities in housing and wealth with Black D.C. residents bearing a disproportionate burden (22). Notably, all of the women in this study identified as Black or African American. This demographic finding is distressingly unsurprising given the long history of structural inequities in D.C., including redlining and predatory lending, shortages of affordable housing, and employment disparities that have resulted in racial wealth gaps for Black residents (14, 22, 58). Many participants in this study shared their desire for full time work but were unable to find jobs with adequate flexibility to manage childcare, and/or provide a wage that makes up for loss of benefit eligibility (59). These persistent structural barriers not only constrain economic mobility but also compound housing instability, limiting opportunities to achieve much desired stability.

### 4.5 Implications

*Adopting a Life Course Approach in Housing and Homelessness Policy and Services*

Our findings suggest that housing and homelessness policymakers should use a life course approach that prioritizes policies and programs aimed at preventing and addressing homelessness across the lifespan. This includes a multifocal strategy which addresses the SDOH, the type and availability of housing support, and the provision of targeted services that support unhoused pregnant people and their children to prevent intergenerational homelessness. While shorter-term programs are critical interventions that promote a quick exit from homelessness, families need affordable, stable long-term housing programs that assist with permanent housing and overall stability (60). Housing transitions should be minimized to provide a path of long-term and intergenerational stability for the pregnant, postpartum, and parenting person and their children (61). Adequate training for care coordinators/case managers can help ensure they are connecting pregnant people to available resources (18). Solutions such as cash transfer programs enable people to prioritize their most pressing needs (e.g., transportation, diapers, food); however, cash transfer can be difficult to access and may expose participants to invasive family monitoring and sanctions (62–64). Recommended long-term strategies that provide stability and encourage low income housing in affluent areas include job training and placements, subsidized housing, and policy reforms such as tax incentives. In addition, restructuring low-income community zoning policies would encourage residential and commercial development (65).

Given the strong association between homelessness and mental illness (66), as well as the profound mental health impact from the stress of housing instability during pregnancy, unstably housed pregnant people may benefit from routine universal referrals and warm hand-offs to mental health providers and ongoing mental health resources. Housing service providers should maintain a list of accessible mental health providers who accept public insurance including Medicaid. Mental health services should ideally be offered across multiple modalities, integrating in-person, telehealth, and text-based support services to mitigate barriers to care (57, 60). After mental health care has been established, regular contact with mental health providers may be beneficial for ongoing support. By providing routine mental health services, we acknowledge the circumstances that pregnant, postpartum, and parenting people face are difficult and merit attentive and ongoing care.

All service providers working with pregnant, postpartum, and parenting people experiencing homelessness should be trained in and implement a trauma-informed approach to care. Recent evidence has found an association between more intensive social support and patient education in the prevention and treatment of postpartum depression, such as home visiting, culturally humble nurse support, and peer support (67). Pregnant, postpartum, and parenting people experiencing homelessness deserve respect while seeking services. Respect is demonstrated through friendly, welcoming, and caring attitudes and also by delivering timely, accurate, and consistent support (68). Service providers should foster a care culture in which respect is a requisite component and ensure that the staff have adequate training, support and accountability.

### 4.6 Strengths and limitations

This study had several strengths. The research was a result of a highly collaborative partnership between academic researchers, policy makers across several DC government agencies, and community-based agencies that provide homelessness assistance. This served as a unique opportunity to generate new evidence with the purpose of understanding the lived experience of pregnant people navigating homelessness assistance during the perinatal period. This is critically important as the study population has historically been hidden in research and policy foci. Findings may lead to the generation of resident-informed innovative policy and practice change recommendations to catalyze system level transformation. Additional strengths include the life course perspective in examining homelessness during pregnancy, which allowed for deep reflection and study findings of cumulative adversity across the life span. Furthermore, the study’s action-oriented approach generated critical opportunities for policy and practice reforms.

Our study also had several limitations. While rates of homelessness in the D.C. community mimic national trends, and experiences of homelessness during the perinatal period may be similar across the U.S., the findings of our study may not be transferable to other metropolitan areas. We only collected data from English speakers, and cannot speak to the experiences of non-English speaking people who experienced homelessness during pregnancy in D.C. - a growing population due to the busing of migrants from the Southern border (69). Because data was collected during a time of great flux and uncertainty due to the COVID-19 pandemic, and homelessness assistance promoting earlier access to services from the 3rd to the 1st trimester was implemented during the time of the study, applicability of the findings to other circumstances may be limited. The interview guide did not ask specific questions about the COVID-19 pandemic so findings may not fully capture the impact of the health crisis. The purposive sampling strategy may have introduced selection bias, as participants were not selected randomly but identified through homelessness services agencies. Finally, all 20 study participants identified as Black or African American and as women, thus limiting its generalizability to other racial groups and genders.

## 5 Conclusion

Guided by a life course perspective, this study explored the perinatal mental health impact of D.C. residents who experienced homelessness while pregnant or as new parents during the COVID-19 pandemic. The stories illuminate the dynamic arc of homelessness in the perinatal period, a phenomenon with the potential to exhibit far reaching consequences, impacting the long-term stability of children and families. A life course perspective compels us to consider the ways in which homelessness during this vulnerable time period has far reaching consequences, impacting later life stages of the pregnant, postpartum, and parenting person but also the life trajectories and wellbeing of their children. A trauma-informed and life course approach can help policymakers intervene longitudinally and intergenerationally for those experiencing homelessness during pregnancy. Existing gaps in the homelessness response infrastructure must include strategies that support long-term stability including sustained access to housing, economic support, and accessible mental health services tailored to the needs of pregnant, postpartum, and parenting people.

## Data Availability

The raw data supporting the conclusions of this article will be made available by the authors, without undue reservation.
