# Supplementary material for: Homelessness in pregnancy: life course factors and mental health in the context of COVID-19
Source: Front Psychiatry. 2025 Dec 26;16:1509350. doi: 10.3389/fpsyt.2025.1509350 (PMC12785181; doi:10.3389/fpsyt.2025.1509350)
Supplement: Supplementary file 1 [file DataSheet1.docx]

# **Supplementary Materials**

##

##

S1 - Agencies Contacted for Participant Recruitment

S2 - Qualitative Interview Guide

##

##

##

## **S1 - Agencies Contacted for Participant Recruitment**

● Department of Human Services, Office of Work Opportunity

● Department of Human Services, Virginia Williams Resource Center

● Homelessness Prevention Providers

○ Community of Hope*

○ Everyone Home

○ MBI

● Homeless Services Family providers

○ Community of Hope*

○ Housing Up

○ CORE DC

○ Friendship Place

○ Community Connections

○ Catholic Charities

○ Coalition for the Homeless

● Youth providers

○ Sasha Bruce Youthworks

○ Echelon

○ Covenant House

○ Casa Ruby

○ HER Resiliency Center

○ Latin American Youth Center

○ SMYAL

○ The Wanda Alston Foundation

● Women service providers

○ Calvary Women’s Services

○ House of Ruth

○ New Endeavors by Women

○ N Street Village

● Domestic Violence and homeless services

○ DASH

○ My Sister’s Place

● Non-Continuum of Care Providers (i.e. outside of DC/ HUD funded agencies)

○ Northwest Center

○ Gabriel Network

○ Missionaries of Charity/ Queen of Peace

○ St. Ann’s Infant & Maternity Home

○ Healthy Babies Project

● Other providers

○ Salvation Army

○ Edgewood Brookland Family Support Collaborative

○ Covenant House

○ Georgia Avenue Family Support Collaborative

○ Veterans on the Rise

○ East River Family Strengthening Collaborative

## **S2 - Qualitative Interview Guide**

Welcome. My name is ______. Thank you for coming here today to talk with us. We’ve asked each of you to come here today because you are currently experiencing or you have experienced homelessness during pregnancy. We don’t know enough about the experiences of people who are pregnant and experiencing homelessness, and want to learn more about how to provide better care and services.

Let’s get started.

1. Please tell us about how you ended up experiencing homelessness.
   1. PROBE: Was being pregnant part of the reason why you ended up experiencing homelessness?
2. Please tell us about what it was like to be pregnant while you were experiencing homelessness.
   1. PROBE: What are some of the biggest challenges you experienced when you were experiencing homelessness during pregnancy?
   2. PROBE: For example, did you have any challenges related to prenatal care, accessing ultrasounds/antenatal testing, food, transportation, work, childcare, mental health like anxiety, other medical care
3. What do you think would have been most helpful to you when you were pregnant and experiencing homelessness?
   1. PROBE: Were there any services that were particularly helpful?
   2. PROBE: Were there any services that disappointed you or let you down?
   3. PROBE: Is there something that would have made your experience easier? Like a person to talk to or more information on resources?
4. Some people who experience homelessness in pregnancy may seek out housing support. Did you ever try to access any house support services? What was that like?
   1. PROBE: Please describe the support/assistance you received while in need of help with safe and stable housing.
   2. PROBE: How many weeks pregnant were you?
   3. PROBE: Were you ever sent away?
   4. PROBE: Were there any barriers that stopped you from getting the support you needed?
5. What were some of the ways you were able to get the things you needed while you were pregnant and homeless?
   1. CLARIFICATION: For example, food, clothes, a place to sleep or a phone.
   2. PROBE: Would an app with resources or a person to talk to have been helpful?
6. What did you do to take care of your health and the health of your pregnancy while you were experiencing homelessness?
   1. PROBE: *Many people find it hard to keep prenatal care appointments. Was there anything that made it hard to get appointments scheduled? Or to get to your appointments?*
   2. PROBE: Sometimes doctors or midwives write prescriptions or give referrals to things like sonograms or specialist care. Did you have any experiences like that during your pregnancy? Were you able to access what you needed?
   3. PROBE: What motivates pregnant people to start and keep getting prenatal care?
7. Is there anything you’d like to share or discuss today?

##

## 
